# Comparative analysis of contemporary anti-double stranded DNA antibody assays for systemic lupus erythematosus

**DOI:** 10.3389/fimmu.2023.1305865

**Published:** 2023-12-07

**Authors:** Claus-Juergen Bauer, Pantelis Karakostas, Nadine Weber, Charlotte Behning, Birgit Stoffel-Wagner, Peter Brossart, Ramona Dolscheid-Pommerich, Valentin Sebastian Schäfer

**Affiliations:** ^1^ Department of Oncology, Hematology, Rheumatology and Clinical Immunology, Clinic of Internal Medicine III, University Hospital of Bonn, Bonn, Germany; ^2^ Department of Medical Biometry, Informatics and Epidemiology, University Hospital of Bonn, Bonn, Germany; ^3^ Institute of Clinical Chemistry and Clinical Pharmacology, University Hospital of Bonn, Bonn, Germany

**Keywords:** systemic lupus erythematosus, SLE, dsDNA antibody, dsDNA antibody test, dsDNA antibody assay, double-stranded DNA, autoimmune disorder, immunology

## Abstract

**Objective:**

Elevated double-stranded DNA (dsDNA) antibody levels in blood serum are considered a disease-specific marker in systemic lupus erythematosus (SLE), correlate with disease activity and the incidence of lupus nephritis, and can be detected in up to 86% of all SLE cases. Despite the high clinical relevance, the variety of dsDNA antibody testing methods with heterogenous performance in clinical use remains challenging. This study is the first to prospectively investigate the performance of two of today’s most commonly applied anti-dsDNA testing methods head-to-head under real-world conditions, as well as their correlation with other clinical and serological disease parameters in SLE patients.

**Methods:**

In this prospective study, all SLE patients undergoing treatment at the Department of Rheumatology at the University Hospital Bonn within a 13-months period (n=41) and control patients without connective-tissue disease (n=51) were consecutively enrolled and examined. For all study participants’ serum samples both anti-dsDNA-NcX enzyme-linked immunoassay testing EUROIMMUN, Luebeck, Germany) and the fluorescence immunoassay ELiA dsDNA (Thermo Fisher Scientific, Waltham, USA) were performed. In addition, demographic data, further laboratory values and disease activity parameters were recorded. Clinical disease activity was assessed by SLEDAI-2K.

**Results:**

Both assays showed high specificity (anti-dsDNA-NcX ELISA: 0.9, ELiA dsDNA: 0.959), but there were notable differences in sensitivity (anti-dsDNA-NcX ELISA: 0.51, ELiA dsDNA: 0.38). Pearsons’s correlation yielded a positive correlation between anti-dsDNA concentrations and CRP concentrations for the anti-dsDNA-NcX ELISA (R=0.22; p=0.038) and a mild-to-moderate inverse correlation between concentrations of anti-dsDNA and complement C4 for the ELiA dsDNA test (R=-0.22; p=0.045) when SLE and control patients were considered together. Other than, no significant correlation between anti-dsDNA concentrations and clinical or laboratory findings was found for either test procedure.

**Conclusion:**

Both anti-dsDNA antibody assays represent reliable examination methods with high specificity for the diagnosis of SLE that fulfill EULAR/ACR requirements. However, the anti-dsDNA-NcX ELISA showed superior sensitivity and significant correlation with disease activity (as measured by CRP concentrations).

## 1 Introduction

Systemic lupus erythematosus (SLE) is a chronic systemic autoimmune disorder with variable organ manifestation and severity in course. Reported incidence and prevalence rates vary geographically and over time but there are estimated to be up to 241 existing (1) and 0.3 - 23.2 (but on average two to five) new cases per 100,000 citizens in the western world (2). The disease is characterized by antinuclear antibodies in 95-100% of all patients (3, 4), subclassifying into autoantibodies against a wide range of self-antigens (5). Elevated double-stranded DNA (dsDNA) antibody levels in blood serum account for a disease-specific marker and can be detected in up to 86% of SLE cases (6). DsDNA antibodies play an essential role in the diagnosis, classification, and management of SLE patients, as they are part of the ACR/EULAR classification criteria (7), tend to correlate with disease activity (8), serve as a predictor of disease flares (8) and are associated with lupus nephritis (9), one key driver of morbidity and mortality in SLE patients (10).

Throughout the last decades, multiple assay methods have been developed and applied in measuring anti-dsDNA which mainly vary in the manner of DNA presentation (serving as the test’s antigen) and the spectrum of antibodies detected. Considering the jungle of dsDNA antibody test systems, three main methodologies stand out and have been established sustainably: radioimmunoassays, Crithidia luciliae immunofluorescence assays and enzyme-linked immunosorbent assays (11). First described in 1969 (12), anti-dsDNA antibodies were initially investigated by Farr radioimmunoassay, utilizing radiolabeled dsDNA for autoantibody detection and quantification. This method is characterized by its particular detection of high-avidity autoantibodies, which are considered to play an extraordinarily important role in the pathogenesis of SLE (13). In further development of this approach, the polyethylene glycol (PEG) precipitation assay was created, a radioimmunoassay with modifications to also include the detection of low avidity anti-dsDNA antibodies (14). The next evolutionary step aimed at the elimination of radioisotope utilization and gave rise to the Farr fluorescent immunoassays [Farr-FIA (15)] and the Crithidia luciliae immunofluorescent test (CLIFT). Finally, the trinity of anti-dsDNA assay methodologies was completed with the establishment of enzyme-linked immunosorbent assays (ELISA), which have evolved to be today’s most commonly used anti-dsDNA testing method (16). The introduction of ELISA tests overcame the CLIFT assay’s ultimate necessity of well-trained technicians and high workload (17), and instead promoted automatization along with decreased processing time (18).

Despite several decades of research efforts, there is still no consensus on the ideal test system to be used clinically. In the literature, remarkable variability regarding sensitivity and specificity among dsDNA antibody testing methods has been reported (19). The commercial variants of anti-dsDNA ELISA tests use different antigens with different avidities. Therefore, even performance of different anti-dsDNA ELISA tests - although based on the same fundamental technology - may not be comparable (19). This observation has also initiated the discussion whether some test systems (with superior specificity) should be used preferably for screening and first diagnosis, while others (with superior sensitivity) should then be used for follow-up assessments and flare detection (8, 16, 20, 21). Since many anti-dsDNA tests (depending on the components used by the manufacturer) detect different anti-dsDNA subpopulations (18, 22) and may detect not only autoantibodies directed against dsDNA but also against other antigens (23, 24), and since autoantibodies detectable in SLE patients differ with respect to their associated SLE manifestations and clinical course (16, 25) (e.g. association with lupus nephritis (26)), additional confusion arises even in the case of a positive dsDNA antibody test result due to the question of what clinical significance should be attached to the positive dsDNA antibody result depending on the test system used (22).

In order to at least reduce false positive or negative test results, manufacturers have pursued various strategies of assay design optimization or component adaptions. For example, with regards to ELISA technology application, a major deterioration factor of test accuracy had usually been that linkers to attach dsDNA to the plate (most commonly used: protamine sulfate or poly-L-lysine) caused nonspecific reactions and antibody binding (27). As a solution to this issue, Thermo Fisher Scientific designed their “ELiA™ dsDNA” (ELiA= enzyme-labelled anti-isotype assay) to be free of any components that could cause false antibody binding (28) and only consist of the target antigen (double-stranded DNA in plasmid form) coated to the well walls (29). In contrast, EUROIMMUN decided to stick with a linker in the design of their “anti-dsDNA-NcX ELISA” (an acronym for anti-double-stranded DNA nucleosome-complexed ELISA) but applied only highly-purified nucleosomes that are free from histone H1, Scl-70 and other non-histone proteins as linkers (27).

This manuscript presents the first study to prospectively investigate the performance of ELiA dsDNA (Thermo Fisher Scientific) and anti-dsDNA-NcX ELISA (EUROIMMUN) – two of the most commonly applied anti-dsDNA antibody tests (16) – head-to-head under real-world conditions and also considering their correlation with other disease-specific parameters. In contrast to previously published reports, this study systematically enrolled all consecutive SLE patients within a single-center approach over 13 months and thereby aimed at assessing the entire local SLE population without any pre-selection bias.

## 2 Materials and methods

### 2.1 Study design and population

This prospective single-center study was performed at the Department of Rheumatology, University Hospital Bonn, Germany. The study followed the principles of the Declaration of Helsinki and the Guidelines of Good Clinical Practice and received ethical approval by the local ethics committee (IRB No. #260/19).

From January 2020 to February 2021, all inpatients and outpatients were prospectively screened, and found to be eligible for study participation when being adult (>18 years) and having a (pre-diagnosed or first diagnosed) confirmed diagnosis of systemic lupus erythematosus according to SLICC criteria (30) and 2019 EULAR/ACR classification criteria for systemic lupus erythematosus (7). Individuals without diagnosis of systemic lupus erythematosus or any other rheumatological disorder were considered eligible as control group participants. The exclusion criteria for both groups encompassed the following points: presence of another connective tissue disease or rheumatological disorder, ongoing inflammation, infection or neoplastic condition. All participants were prospectively enrolled in this study after giving written informed consent.

At baseline, patient characteristics (age, sex, weight, height and body mass index) and relevant standard laboratory values (including hemoglobin, number of leukocytes, thrombocyte count, C-reactive protein concentrations, ANA titer and fluorescence pattern, anti–double-stranded DNA antibody concentrations, concentrations of complement C3 and C4, proteinuria and presence of acanthocytes in urine) were collected. Detailed clinical data regarding the patient history was obtained from Dedalus ORBIS™.

Disease activity assessments were conducted using the Physician Global Assessment (PGA) and SLEDAI-2k. PGA was performed by a board-certified physician. Like most former SLE studies in the literature (31), this study made use of a 0–3 scale for PGA. Score terminology, as used in the “Results” section of this manuscript, was adopted from literature definitions (31, 32).

SLEDAI-2K assessment rates the severity of SLE on a continuous scale, theoretically ranging from 0 to 105, but it has also been validated as a categorical scale [a SLEDAI-2K score of less than three has been validated to reflect a mild condition, a score of 3-6 indicates a moderate condition, and a score of greater than six suggests a severe condition (33, 34)]. This study collected SLEDAI-2k data for all SLE patients not only as present at study visit but also upon initial disease manifestation.

No follow-up visits were conducted as part of this study.

### 2.2 Laboratory analysis

Routinely drawn blood samples were immediately transported to the central laboratory at the University Hospital Bonn which is accredited according to DIN EN ISO 15189. Serum CRP was analyzed by fully automated turbidimetric immunoassay on a cobas® c702 analyzer (Roche Diagnostics, Mannheim, Germany) according to the manufacturer´s instructions (Roche Diagnostics). The sensitivity of the assay was 0.3 mg/l. The reference range was < 0.3 mg/l. The coefficients of variation for intra-assay and inter-assay precision were 2.68% and 2.83% (n=20, mean =5.92 mg/l).

Serum C3c and serum C4 were analyzed by fully automated turbidimetric immunoassay on a cobas® c502 analyzer (Roche Diagnostics) according to the manufacturer´s instructions (Roche Diagnostics). The sensitivity of the assay was 0.04 g/l for C3c and 0.02 g/l for C4. The reference ranges were 0.9 – 1.8 g/l for C3c and 0.1 – 0.4 g/l for C4. The coefficients of variation for intra-assay and inter-assay precision were 0.93% and 2.14% for C3c and 1.11% and 2.62% for C4, respectively.

ANA titers and fluorescence patterns were investigated by using an ANA IIF assay with Mosaic HEp20-10 slides with the EURO Pattern microscope (EUROIMMUN, Luebeck, Germany). Interpretation was done using the EUROLabOffice software (Euroimmun) by two experienced independent investigators. The reference range was <1:80.

**Figure 1:**
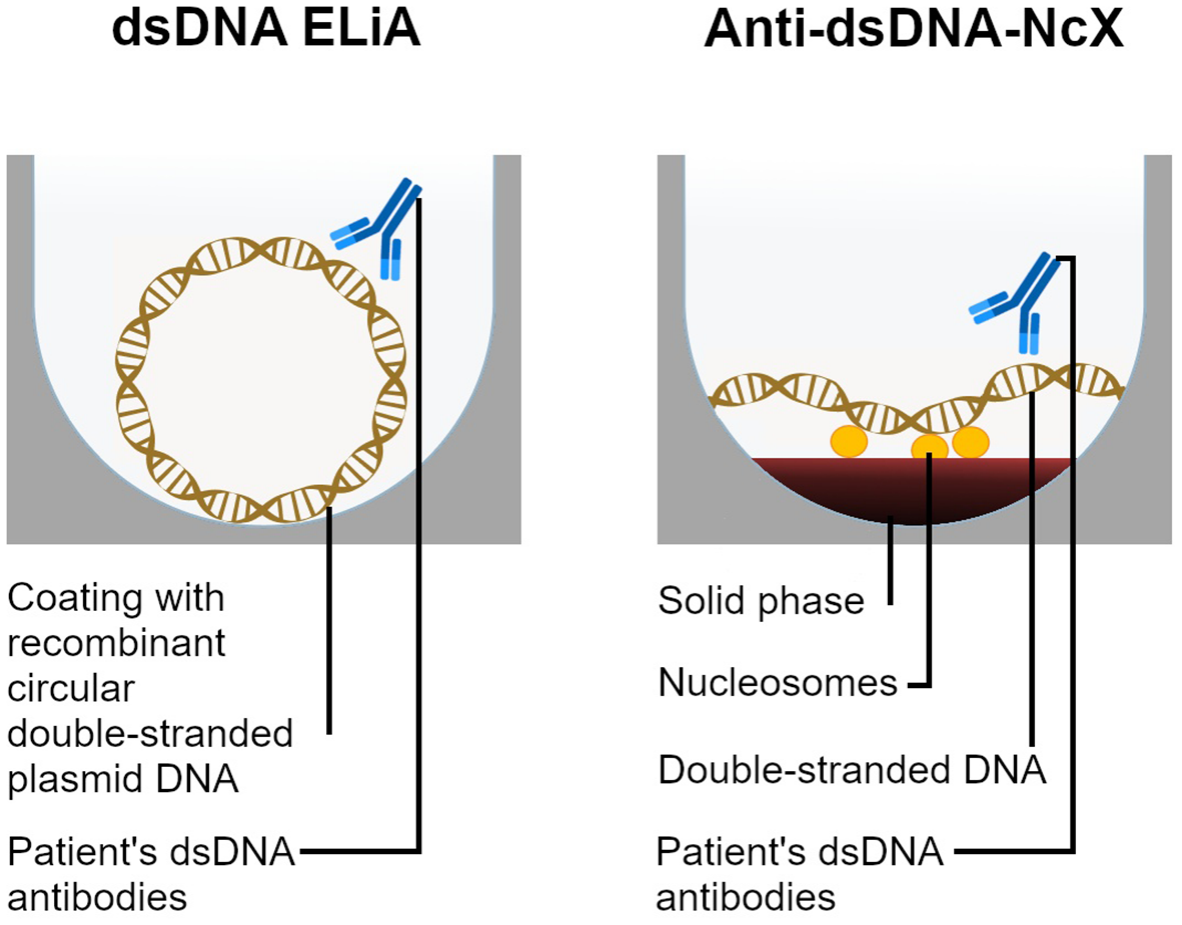
Diagrams and comparison of both dsDNA antibody detection methods applied in this study. ELiA dsDNA wells are coated with circular recombinant plasmid dsDNA in absence of any linker. In contrast, the anti-dsDNA-NcX’s antigen substrate consists of dsDNA complexed with nucleosomes which are linked to the solid phase. Both assays solely detect isotype IgG anti-dsDNA antibodies. Patient’s dsDNA antibody binding is quantified through a fluorescence reaction (ELiA dsDNA) respectively a color reaction (anti-dsDNA-NcX) [not illustrated].

For all samples, two anti-dsDNA assays were conducted. Anti-dsDNA-NcX enzyme-linked immunoassay testing was performed on the EUROIMMUN analyzer I system (EUROIMMUN, Luebeck, Germany). Here, the antigen substrate of the anti-dsDNA-NcX consists of highly purified native dsDNA (origin: salmon testes) complexed with nucleosomes which are coupled to the solid phase (as illustrated in **Figure 1**). The sensitivity of the assay was 2.6 IU/ml. The reference range was <100 IU/ml as suggested by the assay manufacturer based on internal validation with 400 samples from healthy controls that were all negative at this cut-off. The coefficients of variation for intra-assay and inter-assay precision were 3.83% and 4.41%.

Further, fluoroenzyme immunoassay ELiA dsDNA™ was performed on the Phadia® 250 system (Thermo Fisher Scientific, Waltham, USA). The ELiA dsDNA wells were coated with circular recombinant plasmid dsDNA. The sensitivity of the assay was 0.5 IU/ml. The reference range was <15 IU/ml as suggested by the assay manufacturer based on internal validation with 400 samples from healthy controls of Caucasian ethnicity and equal distribution by age and gender that were less than 1% positive at this cut-off. The coefficients of variation for intra-assay and inter-assay precision were 4.82% and 5.21%.

Both assays solely detect isotype IgG anti-dsDNA. The Anti-dsDNA-NcX enzyme-linked immunoassay and the fluoroenzyme immunoassay ELiA dsDNA are calibrated against the international standard Wo/80 (35).

All tests were performed in accordance with the RiliBÄK guidelines of the German Medical Association for the Quality Assurance of Laboratory Medical Examinations and met all criteria for stipulated internal and external quality controls.

### 2.3 Statistical analysis

Statistical analyses were conducted using SPSS ® Statistics (Version 26.0.0.0; IBM Corp., Armonk, USA) and R (Version 4.3.0; R Foundation for Statistical Computing, Vienna, Austria) (36). Descriptive statistics and explorative data analysis were used. Mean, standard deviation, median and ranges were calculated for metric parameters. Categorical data were summarized by absolute and relative frequencies. Correlations between the level of anti-dsDNA concentrations (in each of the two different assays) with clinical and serological disease activity parameters were investigated by Pearson’s correlation coefficient. Additionally, the association between clinical and serological disease activity parameters (including SLEDAI-2k, CRP concentrations and complement C3 and C4 concentrations) as dependent variables and the level of (each assay’s) anti-dsDNA concentrations as independent variables was assessed by separate linear regression model analysis for each group (control, SLE and both groups combined). The threshold for statistical significance was set at p=0.05.

## 3 Results

### 3.1 Patient characteristics

In this study, a total of 41 patients with systemic lupus erythematosus (SLE), and 51 control patients without signs, symptoms or diagnosis of any connective tissue disease were prospectively investigated. The control group consisted of patients who presented for evaluation of a rheumatological disorder but showed no evidence of a rheumatological disease after thorough clinical work-up. Instead, subsequent diagnoses included generalized arthrosis (n=16), finger polyarthrosis (n=14), non-specific arthralgias/myalgias (n=9), psoriasis vulgaris without evidence of psoriatic arthritis (n=7), hypermobility syndrome (n=4), and primary biliary cirrhosis (n=1).

Demographic data and SLE-specific patient characteristics are listed in **Table 1** for both patient groups. Age, weight, height, body mass index and sex are closely matched between both groups. ANA titer and fluorescence pattern distribution across the SLE patient group is reflected by **Supplementary Table 1**. Two SLE patients were enrolled and sampled at the time of diagnosis, further two SLE patients were first diagnosed within the previous 12 months and 37 patients had longer established SLE disease (Duration of disease at the moment of sampling: Mean=76 months, median=111.9 months). Most frequently, patients were on conventional synthetic disease-modifying anti-rheumatic drug therapy (82.9%) and median treatment duration on the current medication was 13 months ( ± 23.04 months as standard deviation) at the time of study baseline. Detailed information regarding treatments administered and treatment durations across the SLE patient group can be found in **Supplementary Table 2**. Physician Global Assessment resulted in “no disease activity” (score=0) in 70.7% of all SLE patients, “mild disease activity” (score=1) in 17.1% of SLE patients, “moderate disease activity” (score=2) in 9.8% of SLE patients, and “severe disease activity” (score=3) in one participating SLE patient (equaling 2.4%).

**Table 1:** Demographic data and core disease-specific patient characteristics.

	SLE Group	Control Group	Entire Study Population
	(n=41)	(n=51)	(n=92)
Age			
Mean (SD)	46.7 (17.2)	46.4 (16.1)	46.5 (16.5)
Median [Min, Max]	46.0 [20.0, 75.0]	45.0 [19.0, 78.0]	45.0 [19.0, 78.0]
Height [meters]			
Mean (SD)	1.64 (0.08)	1.70 (0.09)	1.68 (0.09)
Median [Min, Max]	1.64 [1.50, 1.86]	1.69 [1.53, 1.99]	1.67 [1.50, 1.99]
Weight [kilograms]			
Mean (SD)	66.9 (16.6)	74.0 (20.7)	70.8 (19.2)
Median [Min, Max]	65.0 [44.0, 118]	70.0 [44.0, 176]	68.0 [44.0, 176]
Sex			
Male	7 (17.1%)	15 (29.4%)	22 (23.9%)
Female	34 (82.9%)	36 (70.6%)	70 (76.1%)
Body Mass Index [kg/m²]			
Mean (SD)	24.7 (5.56)	25.2 (5.46)	25.0 (5.48)
Median [Min, Max]	24.0 [18.1, 46.1]	24.6 [17.6, 51.4]	24.5 [17.6, 51.4]
Physician global assessment (PGA)			
Score=0	29 (70.7%)	45 (88.2%)	74 (80.4%)
Score=1	7 (17.1%)	5 (9.8%)	12 (13.0%)
Score=2	4 (9.8%)	1 (2.0%)	5 (5.4%)
Score=3	1 (2.4%)	0 (0.0%)	1 (1.1%)
ELiA dsDNA [IU/ml]			
Mean (SD)	25.3 (44.9)	3.27 (5.23)	13.3 (32.3)
Median [Min, Max]	6.35 [0.600, 213]	1.50 [0.250, 27.0]	2.10 [0.250, 213]
Missing	3 (7.3%)	6 (11.8%)	9 (9.8%)
Anti-dsDNA-NcX ELISA [IU/ml]			
Mean (SD)	413.26 (1076.9)	39.25 (88.5)	205.9 (740.9)
Median [Min, Max]	165.9 [5.0, 5829.9]	5.0 [5.0, 458.7]	16.0 [5.0, 5829.9]
Missing	0 (0%)	1 (1.9%)	1 (1.1%)
SLEDAI-2k score			
Mean (SD)	3.20 (3.04)	NA (NA)	3.20 (3.04)
Median [Min, Max]	2.00 [0, 12.0]	NA [NA, NA]	2.00 [0, 12.0]
Missing	0 (0%)	51 (100%)	51 (55.4%)
CRP level [mg/L]			
Mean (SD)	5.17 (8.31)	4.58 (7.12)	4.85 (7.63)
Median [Min, Max]	2.10 [0.150, 33.3]	1.76 [0.150, 47.4]	2.07 [0.150, 47.4]
Complement component C3 [g/L]			
Mean (SD)	1.03 (0.284)	1.15 (0.351)	1.09 (0.327)
Median [Min, Max]	1.00 [0.420, 2.13]	1.16 [0.190, 2.10]	1.07 [0.190, 2.13]
Complement component C4 [g/L]			
Mean (SD)	0.161 (0.0780)	0.201 (0.0831)	0.183 (0.0829)
Median [Min, Max]	0.150 [0.0200, 0.400]	0.210 [0.0200, 0.410]	0.185 [0.0200, 0.410]
Thrombocyte count [G/L]			
Mean (SD)	226.32 (81.21)	261.29 (69.32)	245.71 (76.85)
Median [Min, Max]	205 [110, 548]	254 [93, 434]	243 [93, 548]
Leucocyte count [G/L]			
Mean (SD)	5.81 (2.03)	7.03 (2.68)	6.49 (2.49)
Median [Min, Max]	5.47 [2.51, 11.09]	6.92 [1.99, 17.91]	6.33 [1.99, 17.91]

[Local laboratory reference ranges:

ELiA dsDNA: negative: <10 IU/ml; equivocal: 10-15 IU/ml; positive: >15 IU/ml.

Anti-dsDNA-NcX ELISA: negative: <100 IU/ml.

C-reactive protein: 0-3mg/L.

Complement component C3: 0.9-1.8 g/L.

Complement component C4: 0.1-0.4 g/L.

Thrombocyte count: 160-370 G/L.

Leucocyte count: 3.6-10.5 G/L].

NA, Not applicable.

On average, the SLE patients in this study presented with moderate disease activity (mean SLEDAI-2k score was 3.20 ± 3.04 as standard deviation). Four out of 41 SLE patients (9.8%) had severe disease activity at the time of study participation, and 14 SLE patients (34.1%) were in the ‘moderate condition’ category. 23 SLE patients who participated in this study (56.1%) had mild disease activity or were in remission according to their SLEDAI-2K score. Detailed information on the frequency of specific organ manifestations and all laboratory abnormalities relevant to the SLEDAI-2k score among the study population can be found in **Table 2**. As study baseline equaled the timepoint of first diagnosis for several patients, but not for many others (whose SLE had been first diagnosed further in the past), **Table 2** reflects both – the clinical and the laboratory status at study baseline and also at the timepoint of first diagnosis (further in the past than study baseline for some patients). In accordance with the aspired partial or full disease remission under therapy, the frequency of many organ manifestations and laboratory abnormalities reflecting disease activity in SLE patients decreased substantially between the timepoints of first diagnosis and study baseline. This fact can be exemplified by the frequencies of leukopenia (10 out of 41 SLE patients at first diagnosis [24.4%] versus 1 out of 41 SLE patients at study baseline [2.4%]), pleurisy (11 out of 41 SLE patients at first diagnosis [26.8%] versus 1 out of 41 SLE patients at study baseline [2.4%]) or arthritis (18 out of 41 SLE patients at first diagnosis [43.9%] versus 4 out of 41 SLE patients at study baseline [9.8%]) within the study patient cohort.

**Table 2:** Distribution of organ manifestations and laboratory abnormalities relevant to the SLEDAI-2k score within the investigated patient cohort.

	SLE Group (n=41)	SLE Group (n=41)	Control Group (n=51)
	Status at study baseline	Status at first diagnosis	Status at study baseline
Clinical findings			
Seizures	0 [0.0%]	0 [0.0%]	0 [0.0%]
Psychosis	1 [2.4%]	1 [2.4%]	0 [0.0%]
Organic brain syndrome	0 [0.0%]	4 [9.8%]	0 [0.0%]
Visual disturbance	1 [2.4%]	1 [2.4%]	0 [0.0%]
Cranial nerve disorder	0 [0.0%]	1 [2.4%]	0 [0.0%]
Lupus headache	0 [0.0%]	3 [7.3%]	0 [0.0%]
Cerebrovascular accident(s)	0 [0.0%]	0 [0.0%]	1 [2.0%]
Vasculitis	1 [2.4%]	2 [4.9%]	0 [0.0%]
Arthritis	4 [9.8%]	18 [43.9%]	4 [7.8%]
Myositis	1 [2.4%]	4 [9.8%]	0 [0.0%]
Lupus Rash	0 [0.0%]	22 [53.7%]	1 [2.0%]
Alopecia	0 [0.0%]	3 [7.3%]	0 [0.0%]
Mucosal ulcers	2 [4.9%]	7 [17.1%]	0 [0.0%]
Pleuritis	1 [2.4%]	11 [26.8%]	0 [0.0%]
Pericarditis	1 [2.4%]	9 [22.0%]	0 [0.0%]
Fever	0 [0.0%]	3 [7.3%]	1 [2.0%]
**Laboratory abnormalities**			0 [0.0%]
Urinary casts	0 [0.0%]	5 [12.1%]	1 [2.0%]
Hematuria [>5 red blood cells/high power field]	4 [9.8%]	5 [12.1%]	6 [11.8%]
Proteinuria [>0.5gram/24 hours]	6 [14.6%]	13 [31.7%]	2 [3.9%]
Pyuria [>5 white blood cells/high power field]	0 [0.0%]	1 [2.4%]	0 [0.0%]
Low complement [C3 or C4 below the lower limit]	13 [31.7%]	13 [31.7%]	2 [3.9%]
Increased DNA binding [DNA binding above normal range for testing laboratory]	21 [51.2%]	32 [78%]	2 [3.9%]
Thrombocytopenia [<100G/L]	0 [0.0%]	5 [12.1%]	1 [2.0%]
Leukopenia [<3G/L]	1 [2.4%]	10 [24.4%]	0 [0.0%]

As study baseline equaled the timepoint of first diagnosis for some patients (n=2), whereas most patients (n=39) had longer established SLE disease, this table reflects both – the clinical and the laboratory status at study baseline and also at the timepoint of first diagnosis (further in the past than study baseline for some patients).

### 3.2 Assay sensitivity and specificity

As displayed in **Table 3**, both ELiA dsDNA (specificity=0.97 [95%-CI: 0.86-1.00]) and anti-dsDNA-NcX ELISA (specificity=0.90 [95%-CI: 0.78-0.97]) showed high specificity. In terms of sensitivity, the ELiA dsDNA (sensitivity=0.38 [95%-CI: 0.22-0.56]) and anti-dsDNA-NcX ELISA (sensitivity=0.51 [95%-CI: 0.35-0.67]) showed notable differences. Because of logistical constraints, three SLE samples and six control patient samples were not included in the ELiA dsDNA investigation, while one control patient sample was excluded from the anti-dsDNA-NcX ELISA analysis. Borderline measurements with ambiguous assignability to either normal or abnormal titers were excluded from the sensitivity and specificity calculations, as shown in **Table 3** (four samples from SLE patients and two samples from control patients). Off note, these four SLE patients and two control patients who tested borderline in the ELiA dsDNA (and were consecutively excluded from specificity and sensitivity calculations for this assay) tested abnormal or normal in the Anti-dsDNA-NcX ELISA exactly 50% of the time in each group.

**Table 3:** Assay sensitivities and specificities.

ELiA dsDNA			Anti-dsDNA-NcX ELISA		
DsDNA antibody level	SLE	Control	DsDNA antibody level	SLE	Control
Abnormal	13	2	Abnormal	21	5
Normal	21	47	Normal	20	45
Borderline*	4	2	Borderline*	0	0
Missing	3	0	Missing	0	1
**Specificity**	0.97 [95%-CI: 0.86-1.00]		**Specificity**	0.90 [95%-CI: 0.78-0.97]	
**Sensitivity**	0.38 [95%-CI: 0.22-0.56]		**Sensitivity**	0.51 [95%-CI: 0.35-0.67]	

The terms “abnormal”, “normal”, and “borderline” were applied congruently to the local laboratory reference ranges as follows:

ELiA dsDNA: Normal: <10 IU/ml; borderline: 10-15 IU/ml; abnormal: >15 IU/ml.

Anti-dsDNA-NcX ELISA: Normal: <100 IU/ml; abnormal: ≥100 IU/ml.

*Borderline measurements with ambiguous assignability to either normal or abnormal titers were excluded from the sensitivity and specificity calculations.

dsDNA, double stranded DNA.

SLE, systemic lupus erythematosus.

In support of **Table 3**, **Supplementary Figure 1** shows correlation graphs directly comparing the levels of anti-dsDNA reactivity by the ELiA dsDNA versus the Anti-dsDNA-NcX ELISA for each sample.

### 3.3 Correlation of assay-specific anti-dsDNA concentrations with other disease characteristics

**Figure 2:**
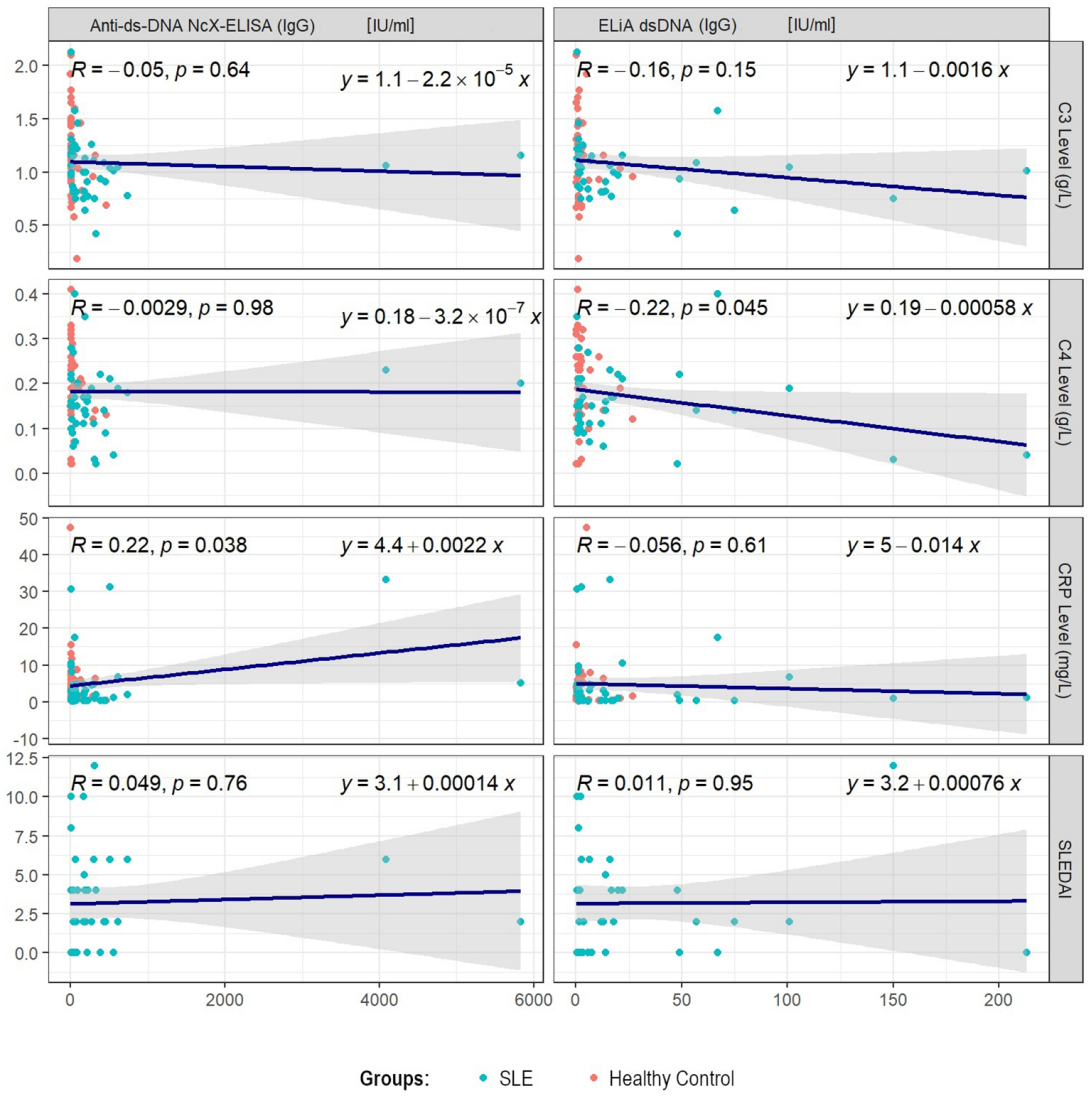
Pearson’s correlations for each assay’s anti-dsDNA titer results and disease activity parameters, such as SLEDAI-2k or serum concentrations of complement C3, C4 or C-reactive protein, when considering SLE and control group patients combined. Each plot illustrates all considered data points, regression line, correlation coefficient R and statistical significance level p in the upper left corner, as well as the mathematical equation of the regression line on the upper right.

**Figure 3:**
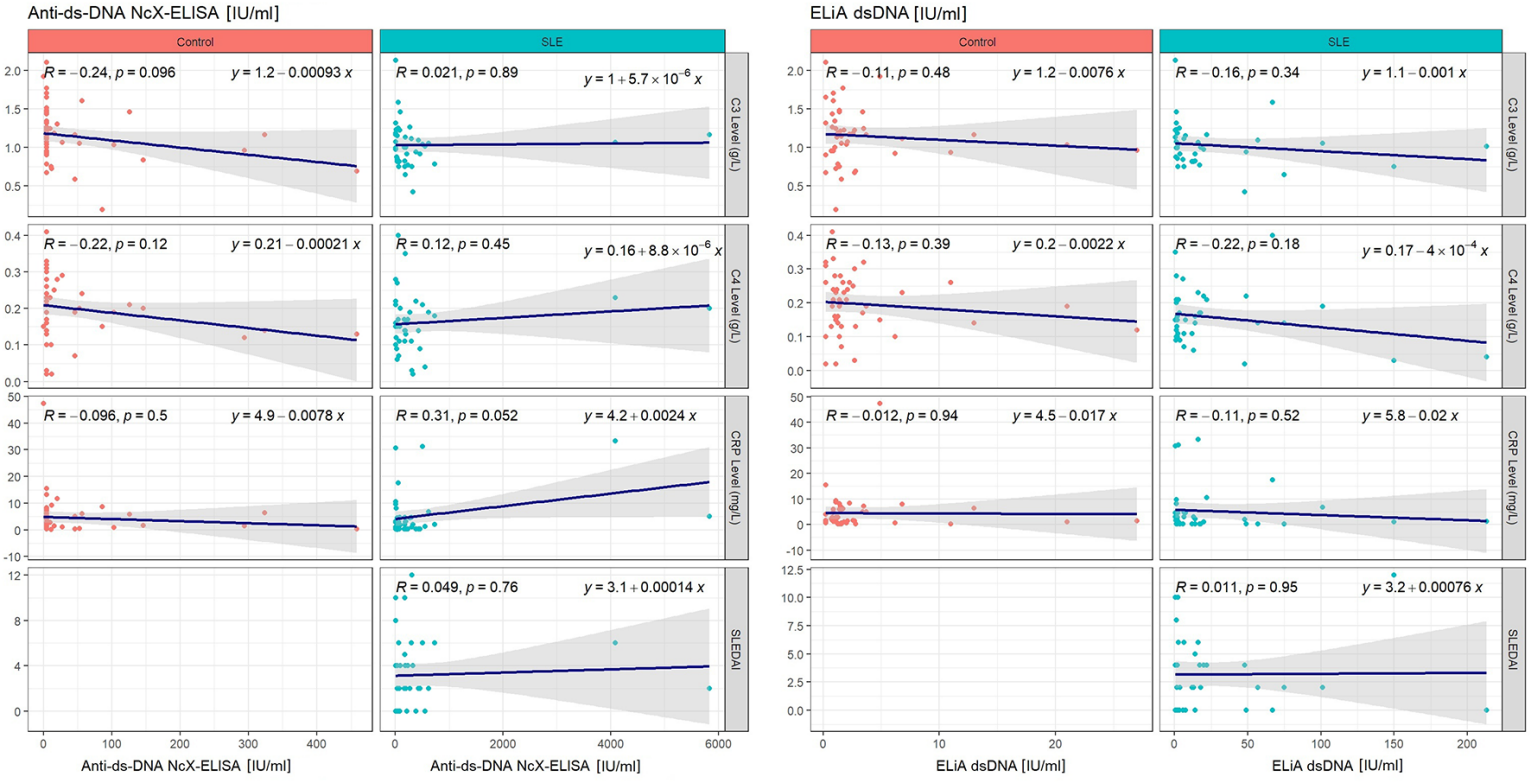
Pearson’s correlations for each assay’s anti-dsDNA titer results and disease activity parameters, such as SLEDAI-2k or serum levels of complement C3, C4 or C-reactive protein, when considering SLE and control group separately. Each plot illustrates all considered data points, regression line, correlation coefficient R and statistical significance level p in the upper left corner, as well as the mathematical equation of the regression line on the upper right. Plots reflecting control group data are indicated by red data points and plots reflecting SLE group data are identifiable through their blue data points. The left half of the Figure only considers the ELiA dsDNA assay, while the right half of the Figure reflects Anti-dsDNA-NcX-ELISA data.

Each assay’s anti-dsDNA concentration results were investigated for correlation with other disease characteristics including concentrations of complement C3 and C4, C-reactive protein and SLEDAI-2k. Analysis was performed for SLE and control group separately, as well as for both groups combined. Pearson’s correlation yielded a mild-to-moderate correlation between concentrations of anti-dsDNA and complement C4 for the ELiA dsDNA test when SLE and control patients were considered together (R=-0.22; p=0.045). Both variables were correlated negatively. Furthermore, Pearson’s correlation showed a positive correlation between concentrations of anti-dsDNA and CRP for the anti-dsDNA-NcX ELISA test when both groups were considered combined (R=0.22; p=0.038). Considering only the SLE patient group, the trend of correlation between anti-dsDNA concentration in the anti-dsDNA-NcX ELISA assay and CRP concentrations persisted but was not statistically significant (R=0.31; p=0.052). All other parameters showed no significant correlation. Detailed information can be found in **Figures 2** and **3** illustrating correlation plots for all relevant parameter combinations. Furthermore, as anti-dsDNA concentrations in SLE have been reported to correlate with lupus nephritis in previous literature, it was investigated to which extent dsDNA concentrations (in each assay) differed between patients with and without the presence of clinical findings relevant to the SLEDAI-2k score that serve as indicators for lupus nephritis and justification to perform a kidney biopsy to confirm/rule out lupus nephritis. While patients with proteinuria in fact presented with higher dsDNA concentrations in both assays and patients with hematuria were found to have higher dsDNA concentrations in the ELiA dsDNA assay compared to patients without hematuria, no statistical significance was reached for either observation. Detailed data is displayed in **Supplementary Table 3**.

### 3.4 Disease activity parameter predictability through anti-dsDNA titers

Linear regression model analysis yielded the following results. Considering the above-mentioned parameter pairs, R² adjusted was low (anti-dsDNA concentration, as measured by ELiA dsDNA, and complement C4 concentrations with SLE and control patients considered together: coefficient estimate=-0.0006, 95%-CI=-0.0012 - -0.0000, p=0.0454, R² adjusted=0.037; anti-dsDNA concentration, as measured by anti-dsDNA-NcX ELISA; CRP concentrations with SLE and control patients considered together: coefficient estimate=0.0022, 95%-CI=0.0001 - 0.0043, p=0.0381, R² adjusted=0.036). Regarding all other parameter pairs consisting of anti-dsDNA concentrations (as measured by each of both assays) as an independent variable and SLEDAI-2k, complement C3, C4 or CRP concentrations as a dependent variable, no relevant associations were found.

## 4 Discussion

In this first prospective study to compare two of the most commonly used commercially available anti-dsDNA tests in SLE head-to-head, we observed excellent specificity of both tests, whereby the anti-dsDNA-NcX ELISA was superior with regards to sensitivity.

To date, no direct comparison of the fluoroenzyme immunoassay ELiA dsDNA (manufacturer: Thermo Fisher Scientific) and the anti-dsDNA-NcX ELISA (manufacturer: EUROIMMUN) with prospective patient enrollment has been performed. Accordingly, most published data available for the ELiA dsDNA is compromised by the fact it was collected in retrospective studies (37–40). After the initial technology validation study (27), the commercially available version of the anti-dsDNA-NcX ELISA was rarely investigated in clinical studies and most of its limited available published data was collected in China (41) and Thailand (42) and solely considered SLE patients of Asian ethnicity.

In comparison to previous studies, the Thermo Fisher Scientific ELiA dsDNA yielded an even slightly higher specificity in our study (specificity=0.97 [95%-CI: 0.86-1.00]). A systematic review published in 2022 (18) reported six [mostly retrospective (37–40)] studies with specificity data regarding the Thermo Fisher Scientific ELiA dsDNA (as of August 2019) with an average specificity of 94.7% [95-CI 91.7%–96.7%]. Of these studies, the ELiA dsDNA’s best performance was observed by Lopez-Hoyos et al. with a published specificity of 96.1% (43). However, this publication leaves unanswered questions, such as the retro- or prospective design of the investigation. Continuing with the above-mentioned systematic review, Carmona-Fernandes et al. (39) have been quoted to report a specificity of 98.1% as they had observed five out of 256 controls with other (non-SLE) rheumatic diseases to be anti-dsDNA positive. However, this quotation neglects the fact that also six out of 100 healthy controls were tested anti-dsDNA positive which needs to be considered as a specificity decrease.

All in all, our study’s results underline that today’s commercially available ELiA dsDNA has overcome common issues of previous plasmid-driven assay generations, such as structural DNA alteration during the coating of the microtiter plates, leading to the exposure of binding sites for anti-single-stranded DNA (anti-ssDNA) antibodies (44, 45) or the occurrence of non-specific bonds with the plastic itself (46) - both resulting in higher numbers of false-positive results.

The anti-dsDNA-NcX ELISA’s specificity in our study (specificity=0.90 [95%-CI: 0.78-0.97]) fits in the middle of three existing previous studies investigating this method, namely Zhao et al., who reported a specificity of 85.0% in Chinese patients (41), Wongjarit et al., who reported a specificity of 96% in Thai patients, and Biesen et al., who found the pre-commercial test version to have a specificity of 98.9% (27). With our study’s specificity result for the ELiA dsDNA and the anti-dsDNA-NcX ELISA’s specificity reported by Biesen et al., both tests have proven to be modern enzyme-linked and fluoroenzyme immunoassays that, in contrast to previously published findings (22), indeed manage to achieve specificity results close to literature-reported specificities of gold standard methods, such as Crithidia luciliae immunofluorescence assays and Farr radioimmunoassays. Thus, both assays also fulfill the requirements for an immunoassay to be eligible for anti-dsDNA antibody testing according to the 2019 EULAR/ACR classification criteria for SLE (“an immunoassay [that has] demonstrated ≥90% specificity for SLE against relevant disease controls”) (7). The fact that the 2019 EULAR/ACR benchmark was introduced also well reflects that, with the growing number and diversity of assays available, standardization has increased in importance. Also for that purpose, the Wo/80 was established (35), an internationally standardized serum for assay calibration (containing solely dsDNA antibodies at a concentration of 200 international units per milliliter) obtainable from the World Health Organization (WHO). In accordance with the general recommendation to no longer use uncalibrated assays, this study strictly adhered to the application of Wo/80 calibration of both assays.

Despite all standardization efforts, notable confounders for anti-dsDNA testing in general remain and so far have only been taken into account in approximately half of the discussed studies (38, 40, 42, 47). Known external factors that can lead to elevated anti-dsDNA titers include drugs, such as selected antiarrhythmics (e.g. procainamide), antihypertensives (e.g. hydralazine), TNF-α inhibitors and sulfasalazine, inter alia (48), but also viral infections (e.g. Epstein–Barr virus infection) (49) and concomitant diseases like autoimmune hepatitis (50). In this study, we checked in all participating patients for these confounding factors, and confirmed none were present.

Apart from specificity, sensitivity marks the second important performance metric for assays.

Regarding the ELiA dsDNA sensitivity, literature reports show considerable heterogeneity. In five studies (37, 38, 40, 43, 47) a bandwidth from 26.7% (43) up to 93% (38) was reported. A major explanatory approach for this heterogeneity could be the patient collective studied in each case, considering that two research groups observed remarkable differences in terms of sensitivity when applying the ELiA dsDNA in SLE patients at first visit [81% (40)] and during active disease [93% (38)], in contrast to patients during follow-up [66% (40)] and quiescent disease [41% (38)]. Results from our study are in line with these literature findings, since EliA dsDNA sensitivity was 38% [95%-CI: 0.22-0.56] in SLE patients with mild to moderate disease activity.

With regards to the Anti-dsDNA-NcX ELISA sensitivity, findings of this study (sensitivity=0.51 [95%-CI: 0.35-0.67]) are similar in magnitude to previous publications that reported 54.62% (41) and 60.4% (27) at the manufacturer’s threshold of 100 IU/ml.

In exploration of the general difference in sensitivity between EliA dsDNA and Anti-dsDNA-NcX ELISA (as observed in our study), main causes may be attributed to the structural differences of both assays. First of all, anti-dsDNA antibodies are a heterogenous group of polyclonal autoantibodies (18) and show different binding patterns depending on whether the dsDNA used as the assay’s antigen substrate is derived from mammalian tissue (e.g., calf thymus), non-mammalian tissue, eukaryotic cells, bacteria, bacteriophages, or even synthetic dsDNA (23, 51). EUROIMMUN’s anti-dsDNA-NcX ELISA uses highly purified native, double-stranded DNA isolated from salmon testes, in contrast to Thermo Fisher Scientific’s ELiA dsDNA that makes use of circular recombinant plasmid dsDNA. Secondly, the dsDNA of the anti-dsDNA-NcX ELISA is not purely and directly applied as a coating to the well’s wall but bound to highly purified nucleosomes. This intends to mimic natural conditions *in vivo* where dsDNA bound to nucleosomes appears on remnants of apoptotic cells that are not timely eliminated and is thus presented to the immune system, serving as a significant B- and T-cell immunogen in patients with SLE (52, 53). Autoantibodies against nucleosomes themselves have been shown to play an important role in the pathogenesis of SLE (54) and be associated with anti-dsDNA antibodies (55, 56). Consequently, the complex of dsDNA and nucleosomes in EUROIMMUN’s anti-dsDNA-NcX ELISA enhances the multispecificity and avidity of the assay and thus could contribute significantly to its increased sensitivity.

These structural differences in both assays possibly explain the positive correlation between concentrations of anti-dsDNA and CRP, which was only revealed by the anti-dsDNA-NcX ELISA but not by the ELiA dsDNA. In general – and maybe also due to the heterogeneity of autoantibodies in SLE, their avidity and frequency of detection in different assays – the connection between anti-dsDNA antibodies and SLE disease activity remains controversial, since persistently elevated anti-dsDNA antibody levels have been observed in SLE patients with disease quiescence, while SLE patients with active disease have also been found to have normal anti-dsDNA antibody concentrations (57). Also, despite its wide clinical application, it is worth noting the complex role of C-reactive protein in SLE, as reviewed by Enocsson et al. (58). Even after anamnestic and clinical ascertainment that patients do not have any active infection or malignancy, as was the case in this study, CRP concentrations and SLE disease activity may still dissociate and challenge the biomarkers reliability in active SLE.

Lastly, we observed a mild-to-moderate negative correlation between concentrations of anti-dsDNA and complement C4 for the ELiA dsDNA test when SLE and control patients were considered together (R=-0.22; p=0.045). Fundamentally, this is in line with previously published reports that described SLE flares to be frequently associated with decreased levels of complement C3 and C4, while anti-dsDNA concentrations increased, particularly in lupus nephritis (59). However, when solely analyzing the SLE patient group, this finding was not confirmed in our study cohort. This result along with the missing correlation between concentrations of anti-dsDNA and complement C3 for the ELiA dsDNA test (when SLE and control patients were considered together) might be attributed to the limited amount of SLE patients with severe disease activity within the investigated patient cohort.

All previously discussed structural and performance disparities between both assays ultimately raise a crucial clinical question: how well do the results from ELiA dsDNA assay and Anti-dsDNA-NcX ELISA even correlate? Initially, our direct comparison of the dsDNA concentration results obtained from each method for every sample (as depicted in **Supplementary Figure 1**, part A) did not yield a significant correlation. However, analysis of the data identified an influential outlier (Anti-dsDNA-NcX ELISA: 4085 IU/ml, ELiA dsDNA: 16 IU/ml). Removing this outlier revealed a statistically significant, moderate positive correlation (refer to **Supplementary Figure 1**, part B). The corresponding patient in fact received in-depth autoimmunserological testing (for study-unrelated clinical concerns) the day of study baseline that showed a strongly positive blot result for anti-nucleosome antibodies, thereby explaining the large discrepancy between both dsDNA assay results.

Beyond the small number of SLE patients with severe disease activity in this study, limitations include the total number of patients enrolled in this study (as a result of the rarity of the disease), as well as the non-longitudinal study design. Furthermore, as Crithidia luciliae immunofluorescence assays (historically known as the gold standard) and modern enzyme-linked/fluoroenzyme immunoassays (being subject to complete automation and easy interpretation) to date still compete in institutional laboratory application, it will be of high clinical significance to correlate both methodologies head-to-head in future studies.

In conclusion, this study prospectively investigated two commercially available variants of two of today’s most commonly used anti-dsDNA testing methods which appeal due to their complete automatability and freedom from radioactive substances. Both assays could be confirmed to be reliable and specific, also fulfilling the anti-dsDNA antibody testing requirements according to the 2019 EULAR/ACR classification criteria for SLE. However, with regards to sensitivity and correlation with CRP, EUROIMMUN’s anti-dsDNA-NcX ELISA showed significant superiority over Thermo Fisher Scientific’s ELiA dsDNA, most likely due to structural assay differences.

## Data availability statement

The raw data supporting the conclusions of this article will be made available by the authors, without undue reservation.

## Ethics statement

The studies involving humans were approved by the local ethics committee (IRB No. #260/19): Ethikkommission der Medizinischen Fakultät Bonn. Building 74, 4th Floor, Venusberg-Campus 1, 53127 Bonn, Germany. The studies were conducted in accordance with the local legislation and institutional requirements. The participants provided their written informed consent to participate in this study.

## Author contributions

C-JB: Data curation, Formal Analysis, Methodology, Project administration, Validation, Visualization, Writing – original draft. PK: Conceptualization, Data curation, Investigation, Methodology, Project administration, Writing – original draft. NW: Data curation, Investigation, Project administration, Writing – original draft. CB: Formal Analysis, Visualization, Writing – original draft. BS-W: Conceptualization, Investigation, Resources, Writing – original draft. PB: Funding acquisition, Resources, Writing – original draft. RD-P: Conceptualization, Investigation, Resources, Validation, Writing – original draft. VS: Conceptualization, Funding acquisition, Investigation, Methodology, Project administration, Resources, Supervision, Validation, Writing – original draft.

## Supplementary material

Supplementary Table 1 ANA titer and fluorescence pattern distribution within the SLE patient group

Supplementary Table 2 Distribution of administered treatments and treatment duration across the SLE patient groups. The table’s right half shows corresponding mean dsDNA concentrations (as determined by both assays) of patients in all medication groups and further investigated to which extent the measured levels of dsDNA antibodies in both assays are explainable by the underlying treatment. The analysis of the regression model showed that there was a low level of explainability for both assays’ dsDNA concentrations based on the medication taken by patients. The ELiA dsDNA assay had an R^2^ value of 0.072 while the anti dsDNA NcX ELISA had an R^2^ value of 0.139. This limited explainability could be attributed to the number of patients examined overall and especially in certain medication groups.

Supplementary Table 3 Clinical indicators for lupus nephritis and corresponding dsDNA concentrations. As anti-dsDNA concentrations in SLE have been reported to correlate with lupus nephritis in previous literature, it was further investigated if those observations are consistent with data from this study’s patient cohort. Focus was on all clinical findings relevant to the SLEDAI-2k score that serve as indicators for lupus nephritis and justification to perform a kidney biopsy to confirm/rule out lupus nephritis. While patients with proteinuria also presented with higher dsDNA concentrations in both assays and patients with hematuria were found to have higher dsDNA concentrations in ELiA dsDNA compared to patients without hematuria, no statistical significance was reached for either observation.

Supplementary Figure 1 Correlation graphs comparing anti-dsDNA concentrations as determined by the ELiA dsDNA assay versus the Anti-dsDNA-NcX-ELISA for each sample. Each plot illustrates the considered data points as indicated by the plot’s title, regression line, correlation coefficient R and statistical significance level p in the upper left corner, as well as the mathematical equation of the regression line on the upper right. Red data points reflect control group data and blue data points reflect SLE group data. **Supplementary Figure 1**, part A depicts the direct comparison of the dsDNA concentration results obtained from each method for every sample. As evident from both plots containing SLE patient data, one single outlier in the upper left corner of both plots (Anti-dsDNA-NcX ELISA: 4085 IU/ml, ELiA dsDNA: 16 IU/ml) substantially changed the data results. In consequence a second analysis (**Supplementary Figure 1**, part B) excluded this single observation, thereby yielding a moderate positive correlation of statistical significance.

## References

[1] Ward MM. Prevalence of physician-diagnosed systemic lupus erythematosus in the United States: results from the third national health and nutrition examination survey. J women’s Health (2004) 13:713–8. doi: 10.1089/jwh.2004.13.713 15333286

[2] Rees F Doherty M Grainge MJ Lanyon P Zhang W. The worldwide incidence and prevalence of systemic lupus erythematosus: a systematic review of epidemiological studies. Rheumatology (2017) 56:1945–61. doi: 10.1093/rheumatology/kex260 28968809

[3] Tan EM Cohen AS Fries JF Masi AT Mcshane DJ Rothfield NF. The 1982 revised criteria for the classification of systemic lupus erythematosus. Arthritis Rheumatism (1982) 25:1271–7. doi: 10.1002/art.1780251101 7138600

[4] Tan E Feltkamp T Smolen J Butcher B Dawkins R Fritzler M. Range of antinuclear antibodies in “healthy” individuals. Arthritis Rheumatism (1997) 40:1601–11. doi: 10.1002/art.1780400909 9324014

[5] Sherer Y Gorstein A Fritzler MJ Shoenfeld Y. Autoantibody explosion in systemic lupus erythematosus: more than 100 different antibodies found in SLE patients. Elsevier (2004) 34:501–37. doi: 10.1016/j.semarthrit.2004.07.002 15505768

[6] Nossent J Huysen V Smeenk R Swaak A. Low avidity antibodies to dsDNA as a diagnostic tool. Ann rheumatic Dis (1989) 48:748–52. doi: 10.1136/ard.48.9.748 PMC1003868 2802796

[7] Aringer M Costenbader K Daikh D Brinks R Mosca M Ramsey-Goldman R. 2019 European league against rheumatism/American college of rheumatology classification criteria for systemic lupus erythematosus. Arthritis Rheumatol (2019) 71:1400–12. doi: 10.1002/art.40930 PMC6827566 31385462

[8] Ter Borg E Horst G Hummel E Limburg P Kallenberg C. Measurement of increases in anti-double-stranded dna antibody levels as a predictor of disease exacerbation in systemic lupus erythematosus. Arthritis Rheumatism (1990) 33:634–43. doi: 10.1002/art.1780330505 2346519

[9] Yung S Chan TM. Mechanisms of kidney injury in lupus nephritis–the role of anti-dsDNA antibodies. Front Immunol (2015) 6:475. doi: 10.3389/fimmu.2015.00475 26441980 PMC4569852

[10] Almaani S Meara A Rovin BH. Update on lupus nephritis. Clin J Am Soc Nephrology: CJASN (2017) 12:825. doi: 10.2215/CJN.05780616 PMC5477208 27821390

[11] Hahn BH. Antibodies to DNA. New Engl J Med (1998) 338:1359–68. doi: 10.1056/NEJM199805073381906 9571257

[12] Pincus T Schur PH Rose JA Decker JL Talal N. Measurement of serum DNA-binding activity in systemic lupus erythematosus. New Engl J Med (1969) 281:701–5. doi: 10.1056/NEJM196909252811304 5821163

[13] Wallace DJ Hahn BH. “Chapter 20 - autoantibodies.,”. In: Dubois’ Lupus Erythematosus and Related Syndromes, Eighth Edition. Philadelphia: W.B. Saunders (2013). p. 273–85. doi: 10.1016/B978-1-4377-1893-5.00020-0

[14] Riley RL Mcgrath H Taylor RP. Detection of low avidity anti-DNA antibodies in systemic lupus erythematosus. Arthritis Rheumatism (1979) 22:219–25. doi: 10.1002/art.1780220303 311203

[15] Lakota K Švec T Kveder T Sodin-Šemrl S Žigon P Ambrožič A. Autoantibodies against dsDNA measured with nonradioactive Farr assay—an alternative for routine laboratories. Clin Rheumatol (2019) 38:353–9. doi: 10.1007/s10067-018-4271-3 30203316

[16] Mummert E Fritzler MJ Sjöwall C Bentow C Mahler M. The clinical utility of anti-double-stranded DNA antibodies and the challenges of their determination. J Immunol Methods (2018) 459:11–9. doi: 10.1016/j.jim.2018.05.014 29807021

[17] Damoiseaux J Agmon-Levin N Van Blerk M Chopyak V Eriksson C Heijnen I. From ANA-screening to antigen-specificity: an EASI-survey on the daily practice in European countries. Clin Exp Rheumatol (2014) 32:539–46. 24983380

[18] Orme ME Voreck A Aksouh R Schreurs MW. Anti-dsDNA testing specificity for systemic lupus erythematosus: a systematic review. J Appl Lab Med (2022) 7:221–39. doi: 10.1093/jalm/jfab146 34996090

[19] Kavanaugh AF Solomon DH. American College of Rheumatology *Ad Hoc* Committee on Immunologic Testing Guidelines. Guidelines for immunologic laboratory testing in the rheumatic diseases: Anti-DNA antibody tests. Arthritis Care Res (2002) 47:546–55. doi: 10.1002/art.10558 12382306

[20] Smeenk R Van den Brink H Brinkman K Termaat R Berden J Swaak A. Anti-dsDNA: choice of assay in relation to clinical value. Rheumatol Int (1991) 11:101–7. doi: 10.1007/BF00304496 1754810

[21] Oo WM O’Neill S. Challenges in systemic lupus erythematosus: From bench to bedside. Trans Autoimmun (2023) 293–331. doi: 10.1016/B978-0-323-85831-1.00015-2

[22] Haugbro K Nossent J Winkler T Figenschau Y Rekvig OP. Anti-dsDNA antibodies and disease classification in antinuclear antibody positive patients: the role of analytical diversity. Ann rheumatic Dis (2004) 63:386–94. doi: 10.1136/ard.2003.016303 PMC1754943 15020332

[23] Rouquette A Desgruelles C. Detection of antibodies to dsDNA: an overview of laboratory assays. Lupus (2006) 15:403–7. doi: 10.1191/0961203306lu2324oa 16898173

[24] Wallace DJ Hahn BH. “Chapter 20 - Autoantibodies; Part B.,”. In: Dubois’ Lupus Erythematosus and Related Syndromes, Eighth Edition. Philadelphia: W.B. Saunders (2013). p. 274–8. doi: 10.1016/B978-1-4377-1893-5.00020-0

[25] Andrejevic S Jeremic I Sefik-Bukilica M Nikolic M Stojimirovic B Bonaci-Nikolic B. Immunoserological parameters in SLE: high-avidity anti-dsDNA detected by ELISA are the most closely associated with the disease activity. Clin Rheumatol (2013) 32:1619–26. doi: 10.1007/s10067-013-2330-3 23857662

[26] Smeenk RJ Van Rooijen A Swaak TJ. Dissociation studies of DNA/anti-DNA complexes in relation to anti-DNA avidity. J Immunol Methods (1988) 109:27–35. doi: 10.1016/0022-1759(88)90438-3 3282012

[27] Biesen R Dähnrich C Rosemann A Barkhudarova F Rose T Jakob O. Anti-dsDNA-NcX ELISA: dsDNA-loaded nucleosomes improve diagnosis and monitoring of disease activity in systemic lupus erythematosus. Arthritis Res Ther (2011) 13:1–9. doi: 10.1186/ar3250 PMC3241370 21329504

[28] Villalta D Bizzaro N Corazza D Tozzoli R Tonutti E. Evaluation of a new automated enzyme fluoroimmunoassay using recombinant plasmid dsDNA for the detection of anti-dsDNA antibodies in SLE. J Clin Lab Anal (2002) 16:227–32. doi: 10.1002/jcla.10045 PMC6807855 12357451

[29] Hernando M González C Sánchez A Guevara P Navajo JA Papisch W. Clinical evaluation of a new automated anti-dsDNA fluorescent immunoassay. Clin Chem Lab Med (2002) 40:1056–60. doi: 10.1515/CCLM.2002.185 12476949

[30] Petri M Orbai A-M Alarcón GS Gordon C Merrill JT Fortin PR. Derivation and validation of the Systemic Lupus International Collaborating Clinics classification criteria for systemic lupus erythematosus. Arthritis Rheumatism (2012) 64:2677–86. doi: 10.1002/art.34473 PMC3409311 22553077

[31] Chessa E Piga M Floris A Devilliers H Cauli A Arnaud L. Use of Physician Global Assessment in systemic lupus erythematosus: a systematic review of its psychometric properties. Rheumatology (2020) 59:3622–32. doi: 10.1093/rheumatology/keaa383 32789462

[32] Furie RA Petri MA Wallace DJ Ginzler EM Merrill JT Stohl W. Novel evidence-based systemic lupus erythematosus responder index. Arthritis Care Res (2009) 61:1143–51. doi: 10.1002/art.24698 PMC2748175 19714615

[33] Gladman DD Ibañez D Urowitz MB. Systemic lupus erythematosus disease activity index 2000. J Rheumatol (2002) 29:288–91. 11838846

[34] Polachek A Gladman DD Su J Urowitz MB. Defining low disease activity in systemic lupus erythematosus. Arthritis Care Res (2017) 69:997–1003. doi: 10.1002/acr.23109 27696791

[35] Feltkamp T Kirkwood T Maini R Aarden L. The first international standard for antibodies to double stranded DNA. Ann rheumatic Dis (1988) 47:740–6. doi: 10.1136/ard.47.9.740 PMC1003590 3052322

[36] R Core Team. R: A language and environment for statistical computing (2023). Vienna, Austria: R Foundation for Statistical Computing. Available at: http://www.R-project.org/ http://www.R-project.org/ (Accessed April 17, 2023).

[37] Launay D Schmidt J Lepers S Mirault T Lambert M Kyndt X. Comparison of the Farr radioimmunoassay, 3 commercial enzyme immunoassays and Crithidia luciliae immunofluorescence test for diagnosis and activity assessment of systemic lupus erythematosus. Clinica Chimica Acta (2010) 411:959–64. doi: 10.1016/j.cca.2010.03.016 20303931

[38] de Leeuw K Bungener L Roozendaal C Bootsma H Stegeman CA. Auto-antibodies to double-stranded DNA as biomarker in systemic lupus erythematosus: comparison of different assays during quiescent and active disease. Rheumatology (2017) 56:698–703. doi: 10.1093/rheumatology/kex314 28053277

[39] Carmona-Fernandes D Santos MJ Canhão H Fonseca JE. Anti-ribosomal P protein IgG autoantibodies in patients with systemic lupus erythematosus: diagnostic performance and clinical profile. BMC Med (2013) 11:1–8. doi: 10.1186/1741-7015-11-98 23557114 PMC3616863

[40] Ghirardello A Villalta D Morozzi G Afeltra A Galeazzi M Gerli R. Diagnostic accuracy of currently available anti-double-stranded DNA antibody assays. An Italian multicentre study. Clin Exp Rheumatology-Incl Suppl (2011) 29:50. 21345292

[41] Zhao J Wang K Wang X Li T Guo L Gu L. The performance of different anti-dsDNA autoantibodies assays in Chinese systemic lupus erythematosus patients. Clin Rheumatol (2018) 37:139–44. doi: 10.1007/s10067-017-3771-x 28741087

[42] Wongjarit K Thammacharoenrach N Dityen K Kaewopas Y Kositpesat N Ukritchon S. Determination of specific autoantibodies in patients with systemic lupus erythematosus by Line immunoassay, ELISA and CLIF. Asian Pacific J Allergy Immunol (2023) 41:73–9. doi: 10.12932/AP-301019-0681 32170924

[43] Lopez-Hoyos M Ayerbe I Martinez-Taboada V Bartolome M Blanco R Lopez-Escribano H. Clinical utility of antibodies to double-stranded DNA by a new immunofluorescence test. Lupus (2004) 13:144. doi: 10.1191/0961203304lu511xx 14995011

[44] Peter JB Shoenfeld Y. “DsDNA autoantibodies.,”. In: Autoantibodies. Amsterdam: Elsevier Science B.V (1996).

[45] Stollar BD. Anti-DNA antibodies. Clinics Immunol Allergy (1981) 1:243–60. doi: 10.1016/S0260-4639(22)00026-3

[46] Brinkman K Termaat R Van den Brink H Berden J Smeenk R. The specificity of the anti-dsDNA ELISA: A closer look. J Immunol Methods (1991) 139:91–100. doi: 10.1016/0022-1759(91)90355-J 2040819

[47] Enocsson H Sjöwall C Wirestam L Dahle C Kastbom A Rönnelid J. Four anti-dsDNA antibody assays in relation to systemic lupus erythematosus disease specificity and activity. J Rheumatol (2015) 42:817–25. doi: 10.3899/jrheum.140677 25684763

[48] Borchers AT Keen CL Gershwin ME. Drug-induced lupus. Ann New York Acad Sci (2007) 1108:166–82. doi: 10.1196/annals.1422.019 17893983

[49] Barzilai O Ram M Shoenfeld Y. Viral infection can induce the production of autoantibodies. Curr Opin Rheumatol (2007) 19:636–43. doi: 10.1097/BOR.0b013e3282f0ad25 17917546

[50] Muratori P Granito A Pappas G Pendino GM Quarneti C Cicola R. The serological profile of the autoimmune hepatitis/primary biliary cirrhosis overlap syndrome. Off J Am Coll Gastroenterology| ACG (2009) 104:1420–5. doi: 10.1038/ajg.2009.126 19491855

[51] Cuomo L Vitillo M Della Rocca M Trivedi P. Comparative analysis of three methods in anti-dsDNA antibodies detection: implications for Systemic Lupus Erythematosus diagnosis. Scandinavian J Immunol (2022) 95:e13123. doi: 10.1111/sji.13123 34865261

[52] Bruns A Bläss S Hausdorf G Burmester GR Hiepe F. Nucleosomes are major T and B cell autoantigens in systemic lupus erythematosus. Arthritis Rheumatism (2000) 43:2307–15. doi: 10.1002/1529-0131(200010)43:10<2307::AID-ANR19>3.0.CO;2-J 11037891

[53] Mohan C Adams S Stanik V Datta SK. Nucleosome: a major immunogen for pathogenic autoantibody-inducing T cells of lupus. J Exp Med (1993) 177:1367–81. doi: 10.1084/jem.177.5.1367 PMC2191002 8478612

[54] Su Y Jia R-L Han L Li Z-G. Role of anti-nucleosome antibody in the diagnosis of systemic lupus erythematosus. Clin Immunol (2007) 122:115–20. doi: 10.1016/j.clim.2006.10.003 17085075

[55] Chabre H Amoura Z Piette J Godeau P Bach J Koutouzov S. Presence of nucleosome-restricted antibodies in patients with systemic lupus erythematosus. Arthritis Rheumatism (1995) 38:1485–91. doi: 10.1002/art.1780381015 7575698

[56] Amoura Z Chabre H Koutouzov S Lotton C Cabrespines A Bach J. Nucleosome-restricted antibodies are detected before anti-dsDNA and/or antihistone antibodies in serum of MRL-Mp lpr/lpr and+/+ mice, and are present in kidney eluates of lupus mice with proteinuria. Arthritis Rheumatism (1994) 37:1684–8. doi: 10.1002/art.1780371118 7980678

[57] Reveille J. Predictive value of autoantibodies for activity of systemic lupus erythematosus. Lupus (2004) 13:290–7. doi: 10.1191/0961203303lu1015oa 15230281

[58] Enocsson H Karlsson J Li H-Y Wu Y Kushner I Wetterö J. The complex role of C-reactive protein in systemic lupus erythematosus. J Clin Med (2021) 10:5837. doi: 10.3390/jcm10245837 34945133 PMC8708507

[59] Julkunen H Ekblom-Kullberg S Miettinen A. Nonrenal and renal activity of systemic lupus erythematosus: a comparison of two anti-C1q and five anti-dsDNA assays and complement C3 and C4. Rheumatol Int (2012) 32:2445–51. doi: 10.1007/s00296-011-1962-3 21706294

