# Supplementary material for: Comparative analysis of contemporary anti-double stranded DNA antibody assays for systemic lupus erythematosus
Source: Front Immunol. 2023 Dec 7;14:1305865. doi: 10.3389/fimmu.2023.1305865 (PMC10733465; doi:10.3389/fimmu.2023.1305865)
Supplement: Supplementary Table 1 — ANA titer and fluorescence pattern distribution within the SLE patient group [file DataSheet_1.docx]

Comparative analysis of contemporary anti-double stranded DNA (anti-dsDNA) antibody assays for systemic lupus erythematosus

Claus-Juergen Bauer^1 ✝*^, Pantelis Karakostas^1 ✝^, Nadine Weber^1^, Charlotte Behning^2^, Birgit Stoffel-Wagner^3^, Peter Brossart^1^, Ramona Dolscheid-Pommerich^3 ‡^, Valentin Sebastian Schäfer^1 ‡^

# Supplementary Tables

| **Titers** | **Total** |  | **Pattern** | |  |
| --- | --- | --- | --- | --- | --- |
|  |  | **Speckled** | **Homogenous** | **Nucleolar** | **Mixed pattern** |
| **Total** | **N (%)** | **15 (36.6)** | **14 (34.1)** | **0 (0.0)** | **12 (29.3)** |
| 1:40 | 0 (0.0) | 0 | 0 | 0 | 0 |
| 1:80 | 7 (17.1) | 2 | 1 | 0 | 4 |
| 1:160 | 7 (17.1) | 2 | 4 | 0 | 1 |
| 1:320 | 7 (17.1) | 4 | 0 | 0 | 3 |
| 1:640 | 9 (22.0) | 1 | 6 | 0 | 2 |
| 1:1280 | 6 (14.6) | 4 | 2 | 0 | 0 |
| 1:2560 | 1 (2.4) | 0 | 0 | 0 | 1 |
| 1:5120 | 3 (7.3) | 2 | 1 | 0 | 0 |
| 1:10240 | 1 (2.4) | 0 | 0 | 0 | 1 |

**Supplementary Table 1**. ANA titer and fluorescence pattern distribution within the SLE patient group

| Substance class | Substance | Patients on this substance: N (%) | Duration of current medication intake: Mean (SD) [months] | Duration of any medication intake: Mean (SD) [months]* | ELiA dsDNA: Mean (SD) [IU/ml] | Correlation between medication and dsDNA concentration (ELiA) | Anti-dsDNA-NcX ELISA: Mean (SD) [IU/ml] | Correlation between medication and dsDNA concentration (NcX ELISA) |
| --- | --- | --- | --- | --- | --- | --- | --- | --- |
| **No medication** |  | 6 (14.6%) | 12.0 (18.2) | 60.8 (64.4) | 23.2 (27.4) | p= 0.9149 | 223.32 (169.9) | p= 0.6459 |
| **Corticosteroids** | Prednisolone | 6 (14.6%) | 15.8 (9.20) | 171 (150) | 17.8 (25.1) | p= 0.4177 | 804.6 (1616.3) | p= 0.3488 |
| **csDMARDs** | All | 34 (82.9%) | 21.5 (24.1) | 113 (92.4) | 24.3 (47.4) | p= 0.7595 | 457.4 (1178.4) | p= 0.5693 |
|  | Hydroxychloroquine | 23 (56.1%) | 18.6 (12.4) | 171.3 (149.8) | 31.5 (56.8) | p= 0.4648 | 604.7 (1414.5) | p= 0.2433 |
|  | Methotrexate | 10 (24.4%) | 14.3 (8.0) | 142.0 (121.3) | 33.6 (70.2) | p= 0.6686 | 243.0 (254.2) | p= 0.2418 |
|  | Azathioprine | 10 (24.4%) | 18.9 (18.5) | 69.1 (49.7) | 27.2 (46.6) | p= 0.6451 | 214.7 (242.1) | p= 0.1548 |
|  | Mycophenolate mofetil | 5 (12.2%) | 38.6 (52.6) | 186.4 (99.3) | 19.5 (31.4) | p= 0.8722 | 84.9 (88.0) | p= 0.2690 |
|  | Chloroquine | 3 (7.3%) | 9.7 (7.0) | 90.7 (34.2) | 16.8 (27.0) | p= 0.8995 | 124.4 (178.2) | p= 0.8761 |
| **bDMARDs** | Belimumab | 6 (14.6%) | 36.7 (50.0) | 93.8 (76.8) | 8.34 (7.7) | p= 0.3636 | 225.9 (314.7) | p= 0.8796 |

**Supplementary Table 2**. Distribution of administered treatments and treatment duration across the SLE patient groups. The table’s right half shows corresponding mean dsDNA concentrations (as determined by both assays) of patients in all medication groups and further investigated to which extent the measured levels of dsDNA antibodies in both assays are explainable by the underlying treatment. The analysis of the regression model showed that there was a low level of explainability for both assays’ dsDNA concentrations based on the medication taken by patients. The ELiA dsDNA assay had an R^2^ value of 0.072 while the anti dsDNA NcX ELISA had an R^2^ value of 0.139. This limited explainability could be attributed to the number of patients examined overall and especially in certain medication groups.

*also includes previous therapy regimens

bDMARDs = biologic disease-modifying anti-rheumatic drugs (incorporates belimumab)

csDMARDs = conventional synthetic disease-modifying anti-rheumatic drugs (incorporates hydroxychloroquine, methotrexate, azathioprine, mycophenolate mofetil, and chloroquine)

dsDNA = double stranded DNA

SD = standard deviation

| **SLEDAI-2k-relevant finding at study baseline** | **N (%)** | **ELiA dsDNA**  **[IU/ml]** | |  | **Anti-dsDNA-NcX ELISA [IU/ml]** | |
| --- | --- | --- | --- | --- | --- | --- |
|  |  | **Mean (SD)** | **p** |  | **Mean (SD)** | **p** |
| Urinary casts | 0 (0.0%) | - | - |  | - | - |
| No proteinuria [<0.5gram/24 hours] | 35 (85.4%) | 23.3 (43.2) | 0.538 |  | 176.3 (180.5) | 0.421 |
| Present proteinuria [>0.5gram/24 hours] | 6 (14.6%) | 35.8 (56.5) |  |  | 745.3 (1640.4) |  |
| No hematuria [<5 red blood cells/high power field] | 37 (90.2%) | 23.6 (41.7) | 0.510 |  | 279.1 (695.3) | 0.620 |
| Present hematuria [>5 red blood cells/high power field] | 4 (9.8%) | 39.5 (73.7) |  |  | 155.5 (173.8) |  |

**Supplementary Table 3**. Clinical indicators for lupus nephritis and corresponding dsDNA concentrations. As anti-dsDNA concentrations in SLE have been reported to correlate with lupus nephritis in previous literature, it was further investigated if those observations are consistent with data from this study’s patient cohort. Focus was on all clinical findings relevant to the SLEDAI-2k score that serve as indicators for lupus nephritis and justification to perform a kidney biopsy to confirm/ rule out lupus nephritis. While patients with proteinuria also presented with higher dsDNA concentrations in both assays and patients with hematuria were found to have higher dsDNA concentrations in ELiA dsDNA compared to patients without hematuria, no statistical significance was reached for either observation.

SD = standard deviation

# Supplementary Figures and figure legends


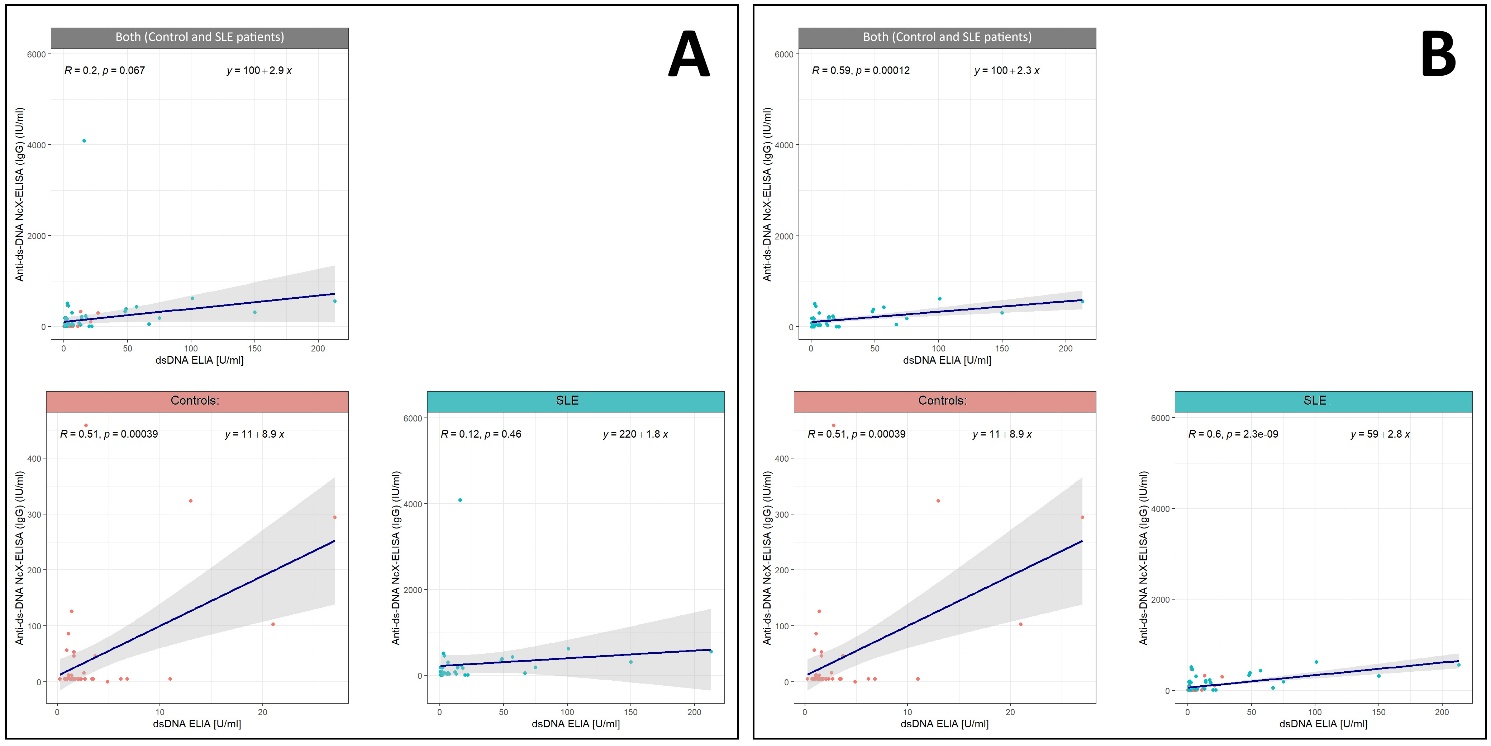


**Supplementary Figure 1.** Correlation graphs comparing anti-dsDNA concentrations as determined by the ELiA dsDNA assay versus the Anti-dsDNA-NcX-ELISA for each sample. Each plot illustrates the considered data points as indicated by the plot’s title, regression line, correlation coefficient R and statistical significance level p in the upper left corner, as well as the mathematical equation of the regression line on the upper right. Red data points reflect control group data and blue data points reflect SLE group data. Supplementary Figure 1, part A depicts the direct comparison of the dsDNA concentration results obtained from each method for every sample. As evident from both plots containing SLE patient data, one single outlier in the upper left corner of both plots (Anti-dsDNA-NcX ELISA: 4085 IU/ml, ELiA dsDNA: 16 IU/ml) substantially changed the data results. In consequence a second analysis (Supplementary Figure 1, part B) excluded this single observation, thereby yielding a moderate positive correlation of statistical significance.
